# Engineered Exosomes Loaded with miR-563 Inhibit Lung Cancer Growth

**DOI:** 10.1155/2022/6141857

**Published:** 2022-06-06

**Authors:** Weiwei Gao, Nan Yang, Chunyang Yin, Yi Zeng, Xiaoping Zhu

**Affiliations:** ^1^Department of Pulmonary and Critical Care Medicine, Shanghai East Hospital, Nanjing Medical University, Shanghai 200120, China; ^2^Department of Tuberculosis, the Second Hospital of Nanjing, Nanjing University of Chinese Medicine, Nanjing 210003, China; ^3^Department of Cardiothoracic Surgery, Jinling Hospital, Medical School of Nanjing University, Nanjing 210002, China

## Abstract

The malignancy of lung cancer (LC) is serious in the world. Exosomes are well-known natural nanovesicles, which are reported to have the potential to carry functional miRNAs as natural carriers and deliver chemotherapeutic drugs. However, the safety and functions of the engineered exosomes for delivering miRNA for the treatment of LC remain to be evaluated. In this study, we found that miR-563 is related to lung cancer from GeneCard database. RT-qPCR and in situ hybridization (ISH) were used to assess miR-563 expression in clinical samples. We prepared and verified the engineered exosomes loaded with miR-563 both *in vitro* and *in vivo*. *In vitro*, flow cytometry, Western blot, and other experimental methods were performed to evaluate the antitumor effect of miR-563 loaded exosomes. In *in vivo*, the LC mouse model was used to observe the effect of the prepared exosomes. The safety of using this exosomes was accessed by liver function test, hematological analysis, and H&E staining in major organs of the mice. Our findings indicated that the miR-563 loaded engineered exosomes inhibit the A549 cells growth *in vitro*, by inhibiting the tumor cell proliferation, migration, and invasion and promoting apoptosis. In *in vivo*, these engineered exosomes were enriched in tumor tissue after injecting to LC model mice and impacted tumor tissue by inhibiting the tumor volume and tumor weight. Importantly, our study indicated that miR-563 loaded engineered exosomes have the potential for clinical application for LC treatment with acceptable safety profiles. Our findings indicate a novel potential therapeutic target for lung cancer patients by miR-563 loaded engineered exosomes.

## 1. Introduction

Lung cancer (LC) seriously threatens human health [1, 2]. The early diagnosis rate of LC is about 15%, and 75% of patients are diagnosed as late stage or metastasis [3]. Based on the basis of Global Cancer Statistics [4], there were emerging more than 2.09 million of LC, about 11.6% of approximately 18.1 million cancer patients worldwide. In addition, 9.6 million patients died of cancers in 2018, including about 1.76 million patients with lung cancer, accounting for 18.4%. Currently, surgical resection and molecular targeted therapy are the main treatment strategies, but there were only 10–20% patients with LC can survive over 5 years [5–7]. Therefore, it is crucial to elucidate the molecular biological mechanisms underlying LC development, therapeutic targets ,and new treatment methods and drugs.

Micro-RNAs (miRNAs) were classical noncoding RNAs with17–24 nt [8]. MiRNAs is stable *in vivo* due to their filling in microcapsules or exosomes [9]. MiRNAs in exosomes have many potential functions. They can transfer to other cells and organs in the body and have different effects in a new location. They can regulate the proliferation, apoptosis, and chemosensitivity of LC cells. Previous studies have shown that miRNA-153-3p can affect LC development and progression [10]. And, miR-613 has been reported to inhibit the development of various cancers [11–13]. Additionally, other studies reported that miR-613 is an important biomarker in LC [14]and represses the occurrence and development of LC by targeting related genes [15].

Exosomes (Exo) have unique functions, which can be used as a personalized carrier for treating diseases by targeted drug delivery. They are produced in cells and recognized by the immune system as “self,” which improves their stability in serum and helps to deliver signals to target cells. Furthermore, compared with artificial nanocarriers, their circulation time *in vivo* is longer, greatly increasing the chance of this molecule meeting with target cells [16]. The feasibility of exosomes as drug carriers has been proposed and conceptually verified [17]. Nie et al [18] prepared exosomes that target lung cancer cells and used it to deliver miRNA-126 for treatment. These engineered exosomes have potential applications in the future.

In order to find an engineered exosome with clinical potential for the treatment of LC, we obtained the miR-563 from GeneCard by searching the keywords “lung cancer” and verified it in clinical samples. Previous studies found that miR-563 can inhibit the growth of LC [19]. In this study, we encapsulated miR-563 in exosomes and then conducted *in vitro* and *in vivo* to observe its influence on tumors and explore its mechanism. Furthermore, we assessed the safety of the engineered exosome in healthy mice.

## 2. Materials and Methods

### 2.1. Screen the Lung Cancer-Related miRNA

We obtained the LC-related genes from GeneCard (https://www.genecards.org/). We entered the keyword “lung cancer,” and the lung cancer-associated gene symbols were obtained, which contained 1,053 miRNAs. Then, we used the relevance score ≥2.0 as the cutoff value and obtained 330 miRNAs. According to the experimental purposes, and determination by literature search screening and subsequent clinical sample testing, miR-563 with reduced expression in LC was selected as the target miRNA of the present study.

### 2.2. Patients and Tissue Samples

We collected 38 pairs of LC clinical tissues and corresponding normal tissues samples from the Second Hospital of Nanjing (Nanjing, China). RT-PCR and ISH were used to test the miR-563 expression in the clinical samples. The tissues were taken from the patients who underwent LC tissue resection in the Second Hospital of Nanjing. All patients were confirmed by diagnosis histopathology. This study was conducted under the supervision of the Second Hospital of Nanjing after the World Medical Association, and all patients have signed informed consent.

### 2.3. Cell Lines and Culture

A549 cells were cultured in RPMI-1640 (HyClone, USA), and the complete medium contains 10% fetal bovine serum (Merck Millipore, Germany). Then, the cells were put at 37°C and 5% CO_2_ atmosphere.

### 2.4. Extracellular Vesicle Isolation

EVs were isolated by differential ultracentrifugation from cell culture medium. Firstly, low-speed centrifugation was used for the depletion of cells and cell debris (2,000 × *g* for 15 min and 16,000 × *g* for 20 min). Then, pellets were centrifuged again for 90 min at 100,000 × *g* at 4°C. EV solutions were filtered through a 0.22 *μ*m syringe filter for future use.

### 2.5. Exosome Loading

Exosomes (10 *μ*g/mL) were mixed with 400 nM of Cy5-labeled miR-563 in 1 mL phosphate buffered saline (PBS). Then, the mixture was electrophoresed at 400 V, 50 sf, three cycles by 30 ms pulse/2 s pause. Then, the exosome samples were diluted and centrifuged at 110,000× *g* for 70 min.

### 2.6. Cell Uptake of Exosomes

Red fluorescent dye PKH26 (Umibio, UR52302, China) was used to mark the engineered exosomes. A549 cells were coincubated with stained Exo. 24 h later, 4′, 6-diamidino-2-phenylindole (DAPI) was used for marking the nucleus. We observed and recorded the PKH26 in A549 cells in each group by confocal microscope (Nikon, Japan).

### 2.7. A549 Cell Proliferation

CCK-8 was used for the proliferation ability of A549 cells after treated with the Exo [20], and then, the CCK-8 reagent was added to the cell medium and incubated at 37°C for 2 h. Then, the absorbance value of OD450 nm was detected at 0 h, 24 h, 48 h, and 72 h, and a microplate reader (Beyotime Biotechnology, China) was used. EdU experiment was used to visualize the DNA expression of A549 cells (Beyotime Biotechnology, China) and then observed under the fluorescence microscope.

### 2.8. Flow Cytometry

In brief, 5 × 10^5^ cells were collected and suspended in PBS. Then, Annexin-V and propidium iodide were added and incubated for for 20 min. After that, 480 mL binding buffer was supplemented into cell suspension after a 10-min incubation. At last, the cell apoptosis ratio was analyzed by FACS Calibur Flow Cytometer (BD Biosciences, USA).

### 2.9. Reverse Transcription-Quantitative PCR (RT-qPCR)

We collected total RNA in cells and tissues using Trizol reagent (Invitrogen, USA), and the thermoscript RT-PCR synthesis kit (Pittsburgh fermentation company, USA) was used to synthesize the first strand cDNA. The ThermoScript RT-qPCR kit was used. The primers used were as follows (Sangon Biotech, Shanghai): miR-563 forward: 5′-AGGTTGACATACGTTTCCC-3′ and reverse: 5′-TCGTATGCCGTCTTCTGCTTGT-3′; U6 snRNA was used as an internal reference in this manuscript: forward: 5′-GACAATCCTAGACTAGCTTACGA-3′ and reverse: 5′-CATGGCACAAGTCATAAGCA-3′. The relative expression analysis of genes was analysis by the 2^−△△Ct^ method.

### 2.10. *In Situ* Hybridization (ISH)

MiRNA ISH was carried out according to the kit instructions (miRCURY LNA™ microrna ISH Optimization Kit; Exiqon Inc., Vedbaek, Denmark). miR-563 probe: Digoxin-5-GACAATCCTAGACTAGCTTACG-3, Digoxin. U6 probe: Digoxin-5-CACGAATTTGCG TGTCATCCTT-3, and negative control probe: Digoxin-5-AGGTTGACA TACGTTTCCC-3-Digoxin.

### 2.11. Characterization of Exosomes

The exosomes were fixed in 2% paraformaldehyde and 0.1 M phosphate and adsorbed to a 2 Formvar-carbon-coated grid. Exosomes were viewed with a transmission electron microscope using Jeol JEM-1400 (Jeol Ltd., Tokyo, Japan) operating at 80 kV. The size of exosomes was analyzed using a Zetasizer Nano ZS system (Malvern Instruments, Malvern, UK).

### 2.12. Western Blot

The whole extracts were separated by sodium dodecyl sulfate polyacrylamide gel electrophoresis (SDS–PAGE) in an electrophoresis instrument (Bio-Rad, USA) and then transferred to a polyvinylidene difluoride (PVDF) membrane. Next, the membrane was washed in TBS Tween 20 and then incubated with primary antibodies against CD63 (1 : 1000, ab134045, Abcam), TSG101 (1 : 1000, ab125011, Abcam), Bax (1 : 1000, ab32503, Abcam), Bcl-2 (1 : 1000, ab32124, Abcam), cleaved-caspased3 (1 : 1000, ab32042, Abcam), cleaved-caspase9 (1 : 1000, ab2324, Abcam), MMP2 (1 : 1000, ab92536, Abcam), MMP9 (1 : 1000, ab76003, Abcam), and *β*-actin (1 : 1000, ab6276, Abcam). The membrane was washed for 3 times and incubated with the corresponding secondary antibody. The protein bands were observed with ECL chemiluminescence kit (Thermo Fisher Scientific, USA).

### 2.13. Wound-Healing Migration Assay

A549 cells (3 × 10^5^) were incubated to reach 70% confluence. After 24 h treatment with loaded engineered Exo and cultured at 37°C for another 4 h, a scratch wound was created with sterile pipette tip, and the scraped monolayer cell image was recorded.

### 2.14. Transwell Assay

The adjusted cells were added to the upper chamber, and the culture medium was replaced in the lower chamber, and then the cells were incubated overnight in an incubator for 24 h. Cells were fixed for 10 min with 4% paraformaldehyde and stained 30 min with 0.1% crystal violet. Per sample choose five fields were randomly selected to assess cells in each group then were observed under an inverted microscope.

### 2.15. *In Vivo* Visualization of Injected Exosomes

BALB/*c* mice were obtained from the Model Research Animal Center of Nanjing University. All animal experiments were conducted in accordance with the regulations on the administration of experimental animals. Animals used in the experiment and their pain were alleviated as much as possible during the experiment. The animals were raised under the Guidance of the Center for Developmental Biology, and all experiments adopt the scheme approved by the institutional animal care subcommittee of the Second Hospital of Nanjing. To evaluate the biological distribution of miR-563 loaded engineered Exo *in vivo*, about 6–8 weeks female BALB/*c* mice were injected with 1 × 10^6^ A549 cells. Until the tumor volume reaches 400 mm^3^, inject mice with PKH26 labeled exosomes 30 mg via IV routes. Small animal imaging system (Kodak, United States) was used to assess the enrich of Exo.

### 2.16. Histopathology

Tumor tissues and other organ samples were collected and fixed in 4% paraformaldehyde. Histological examination was performed in line with a conventional method. H&E and PCNA staining were performed. The slides were scored and identified in a blinded manner.

### 2.17. TUNEL Assay

One-step TUNEL Apoptosis Assay Kit from Beyotime Biotechnology (Beyotime, China) was used for staining. Cells were exposed to the TM red-labeled dUTP after fixed in PBS with 4% paraformaldehyde. And then, samples were counterstained with DAPI. TUNEL positive nuclei were identified and analyzed by fluorescence microscopy.

### 2.18. Serum Level of ALT/AST/ALP Activity Assay

To assessed liver function, mouse serum was used to detect the enzymatic activities of alanine aminotransferase (ALT), aspartate aminotransferase (AST), and alkaline phosphatase (ALP). The blood of mice was collected in centrifuge tubes. After centrifugation, the serum was collected and stored at 4 degrees for detection as soon as possible. Serum from mice was assayed using ALT, AST, and ALP activity assay kits (St. Louis, USA). The light absorbance was measured at 510 nm with a Multiskan MK3 (Biotek, USA).

### 2.19. Blood Biochemical Examination

Blood Biochemical Examination includes the red blood cell (RBC), white blood cell (WBC), and platelet (PLT) count. Blood samples of mice were collected, and centrifugation was used to harvested serum samples for test.

### 2.20. Statistical Analysis

Data were presented as mean ± standard deviation (SD) from at least 3 independent experiments. GraphPad Prism 5.0 software was used for statistical analysis. *T*-test or one-way analysis of variance was used for comparison between two or more groups. *P* < 0.05 means statistically significant difference.

## 3. Results

### 3.1. miR-563 Is Expressed at Low Level in Clinical Samples of LC

**Figure 1:**
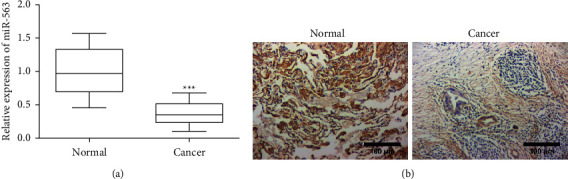
MiR-563 expressed at low level in clinical samples of lung cancer. (a) The mRNA level of miR-563 in normal lung and lung cancer tissue was analyzed by qPCR. Data are mean ± SD from 3 independent experiments (^*∗∗∗*^*P* < 0.001). (b) Representative images of ISH analysis of miR-563 expression in normal lung and lung cancer tissue. Magnification, ×100. Scale bar, 100 *μ*m.

In order to find an engineered exosome with clinical potential for the treatment of LC, we obtained the miR-563 from GeneCard by searching the keyword “lung cancer” and then determined by literature search screening. Subsequently, the clinical sample was tested. We analyzed the lung cancer tissues and adjacent tissues by RT-PCR and ISH for the potential effect of miR-563 in LC. miR-563 mRNA expression was downregulated in the cancer tissues by more than 3-fold compared with the control group (Figure 1(a)). Subsequently, this finding was validated, indicating that miR-563 was generally low expressed in cancer tissue samples (Figure 1(b)).

### 3.2. Preparation and Characterization of miR-563 Loaded Engineered Exosomes

**Figure 2:**
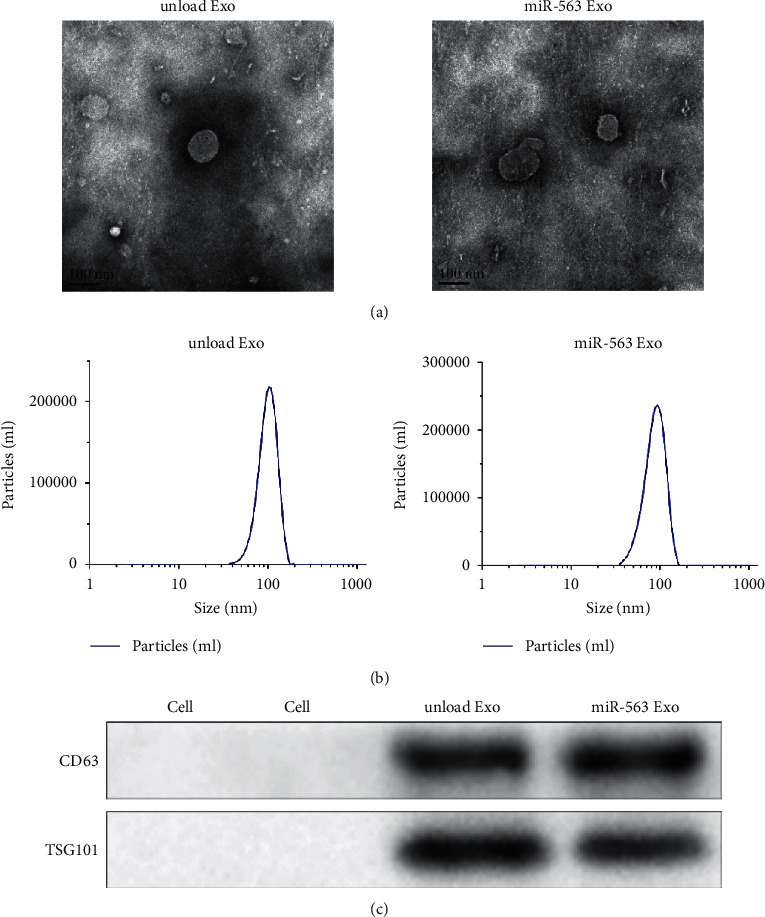
Characterization of miR-563 loaded exosomes. Transmission electron photomicrographs of the unloaded Exo and prepared miR-563 Exo. (b) Representative image of DLS showing the concentration and size distribution of the unloaded Exo and prepared miR-563 Exo. (c) Western blot analysis of exosomal surface markers CD63 and TSG101.

In order to determine the effect of miR-563 in clinical use, we prepared the engineered Exo loaded with miR-563. Transmission electron microscopy (TEM) was used to analyze the prepared engineering Exo. Under electron microscope, these exosomes appeared as disc-shaped with an average particle size of 30–150 nm (Figure 2(a)). Subsequently, DLS analysis was used to assess the average size of Exo. The average size of the miR-563 loaded engineered exosomes was 102 nm (Figure 2(b)). In addition, CD63 and TSG101, which were important extracellular markers of exosomes, were significantly increased (Figure 2(c)). These findings indicate that the engineered Exo loaded with miR563 has been successfully isolated.

### 3.3. Uptake Capability of A549 Cells for the Prepared Exosomes

**Figure 3:**
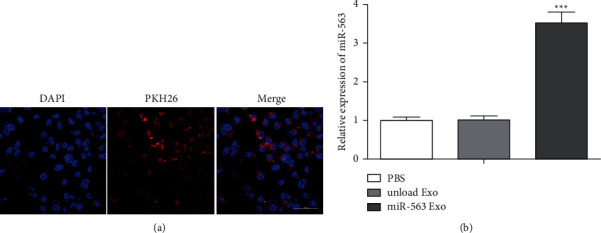
Uptake ability of A549 cells for the engineered exosomes. (a) MiR-563 loaded exosome uptaken by A549 visualized using confocal microscopy. Scale bar = 100 nm. (b) MiR-563 expression was assessed by real-time qPCR.  ^*∗*^ ^*∗*^ ^*∗*^*P* < 0.001*vs*. the unloaded Exo group.

We used the fluorescent dye PKH26 to label Exo, and A549 cells were incubated with the PKH26-miR-563 Exo to verify the specific uptake capability of A549 cells. After ingestion, we first observed the quantity of exosomes getting into cells by assessing the fluorescence intensity. We evaluate the A549 cells' uptake capability to miR-563 loaded engineered Exo. It can be seen from the results that the red fluorescence intensity of the ingestion group is the highest (Figure 3(a)). In addition, RT-PCR analysis showed the miR-563 expression was upregulated in A549 cells treated with miR-563 Exo group (Figure 3(b)). These results indicate that A549 cells have the ability to specifically ingest Exo.

### 3.4. Prepared Exo Inhibits A549 Cell Proliferation and Promotes Apoptosis

**Figure 4:**
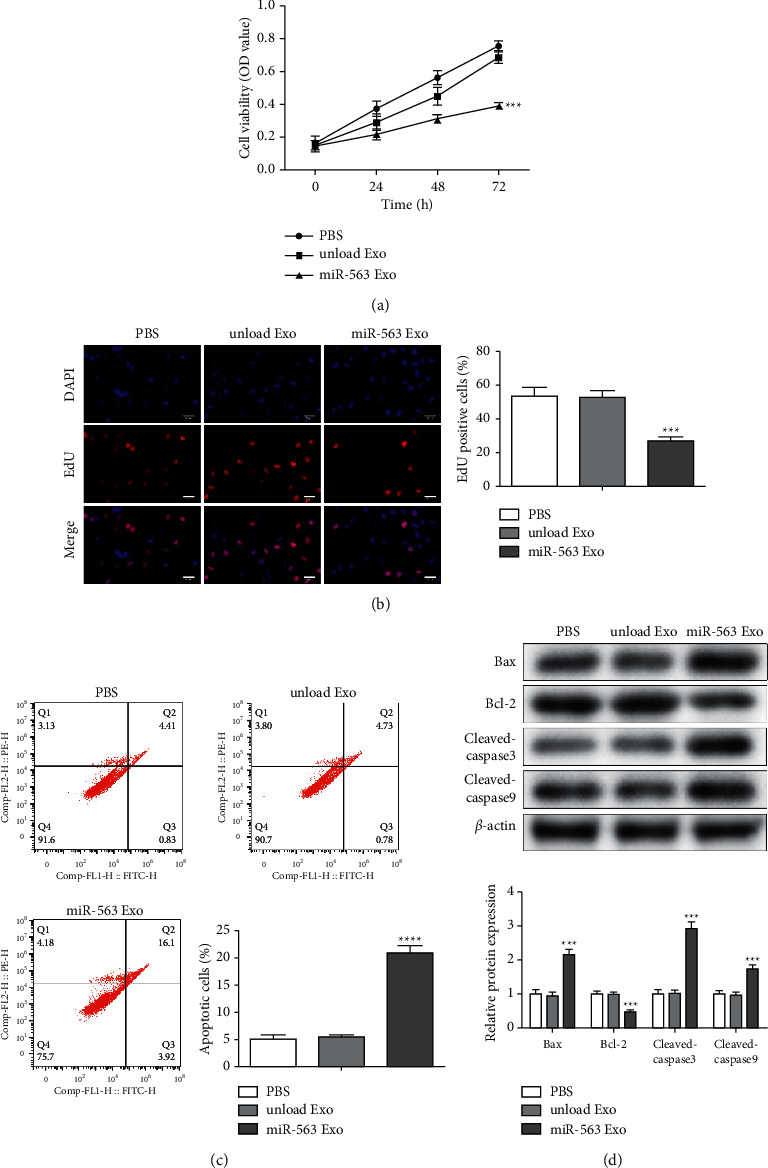
The miR-563 Exo inhibits A549 cells proliferation and promotes apoptosis. (a) The changes in cell viability of A549 incubated with miR-563 Exo for 0, 24, 48, and 72 h was evaluated by CCK-8 assay. (b) EdU staining results of the incubation of A549 cells with exosomes. Scale bar = 100 nm. (c) Flow cytometric detection of apoptosis. (d) The protein expression level of Bax, cleaved caspase-3, cleaved caspase-9, and Bcl-2 evaluated by western blot. The values were presented as mean ± SD (*n* = 3). ^*∗∗∗*^*P* < 0.001 versus the unloaded Exo group.

To explore the role of miR-563 loaded engineered Exo in LC, we first assessed the proliferation and apoptosis of the cancer cells. We investigated the effect of miR-563 loaded engineered Exo on A549 cells proliferation by CCK-8 and EdU analysis. After coincubation with miR-563 Exo for 72 h (Figure 4(a)), which result tested through CCK-8 kit, the cell viability of A549 cells was obviously repressed. Similarly, EdU positive cells ratio of the miR-563 Exo group was significantly decreased (Figure 4(b)). The apoptosis ratio of the miR-563 loaded engineered Exo treatment group was significantly increased (Figure 4(c)) by flow cytometry. In addition, the protein level of Bax, cleaved caspase-3, and cleaved caspase-9 was downregulated almost 3-fold in A549 cells after treatment with miR-563 Exo. Instead, the expression of Bcl-2 was obviously upregulated by about 3-fold (Figure 4(d)).

### 3.5. miR-563 Loaded Engineered Exo Inhibits the Migration and Invasion of A549 Cells

**Figure 5:**
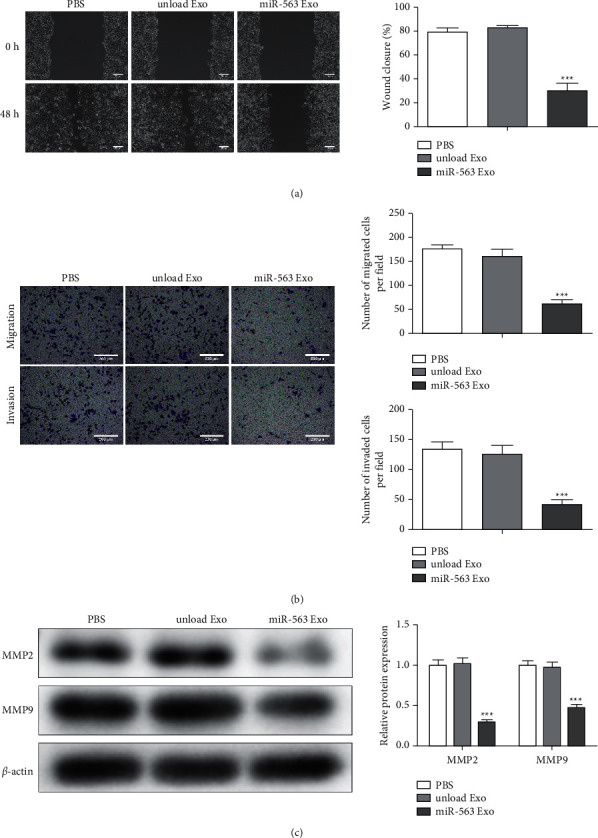
MiR-563 loaded engineered Exo inhibits the migration and invasion of A549 cells. (a) The influence of miR-563 Exo on the migration ability of A549 cells assessed via scratch test. Scale bar = 200 nm (b). Transwell assay evaluated the migration and invasion capacity of A549 treated with miR-563 Exo. Scale bar = 200 nm (c). The protein expression of MMP2 and MMP9 evaluated by western blot. The values were presented as mean ± SD (*n* = 3). ^*∗∗∗*^*P* < 0.001 versus the unloaded Exo group.

We explored whether the miR-563 loaded engineered Exo inhibits the cell migration and invasion of A549 cells. It was found that the wound closure of A549 cells treated with miR-563 Exo was significantly delayed (Figure 5(a)), which indicates that miR-563 loaded engineered Exo inhibited the migration of A549 cells. Besides, transwell analysis of A549 cells demonstrated that there was a significant repression of the invasion and migration of cells in miR-563 Exo-treated treated group than control groups (Figure 5(b)). Furthermore, the protein level of MMP2 and MMP9 was downregulated almost 3-fold in A549 cells after treatment with miR-563 Exo in Figure 4(c). These results suggest that the specific uptake of miR-563 loaded engineered Exo leads to inhibition of the invasion and migration of A549 cells.

### 3.6. miR-563 Loaded Engineered Exo Plays an Antitumor Role *In Vivo*

**Figure 6:**
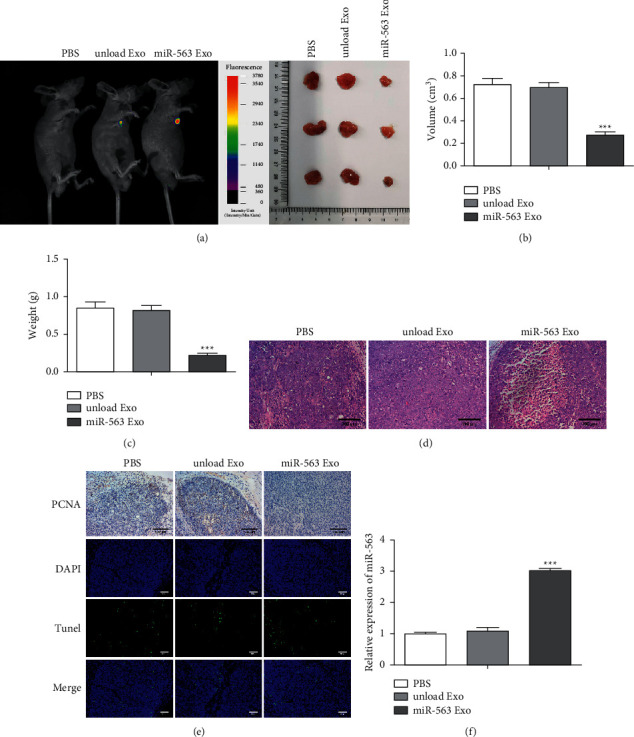
The antitumor role of miR-563 loaded Exo *in vivo.* (a) Imaging of PKH26-labeled exosomes in mice. (b) Tumor images in different groups of mice and the changes in tumor volume of mice treated with different treatments with 16 days. (c) Tumor weight of mice in different groups. (d) H&E of tumor sections. Scale bar, 100 *μ*m. (e). TUNEL staining on tumor sections. Scale bar, 100 *μ*m. (f) MiR-563 expression assessed by real-time qPCR. The values were presented as mean ± SD (*n* = 3). ^*∗∗∗*^*P* < 0.001 versus the unloaded Exo group.

We continued to evaluate the biodistribution and tumor penetration of miR-563-loaded engineered Exo in mice with tumors. miR-563 loaded engineered Exo labeled with PKH26 was easily found in model mice (Figure 6(a)). In Figure 6(b), the tumor volume of the miR-563 Exo group mice was significantly decreased. Similarly, the tumor weight of mice treated with miR-563 Exo was significantly reduced (Figure 6(c)). H&E staining was assessed in tumors from mice and showed that the tumor was obviously increased (Figure 6(d)). Then, we examined whether miR-563 Exo promotes tumor apoptosis by TUNEL staining, which was decreased (Figure 6(e)). Similarly, the levels of apoptosis were increased in the miR-563 Exo group (Figure 6(e)). And, the expression of miR-563 in the tumor was evidently upregulated in Figure 6(f). The above *in vivo* experimental results suggested that miR-563 loaded engineered Exo promoted the apoptosis of tumor cells and exhibited an antitumor effect *in vivo*.

### 3.7. *In Vivo* Safety Evaluation of miR-563 Loaded Engineered Exo

**Figure 7:**
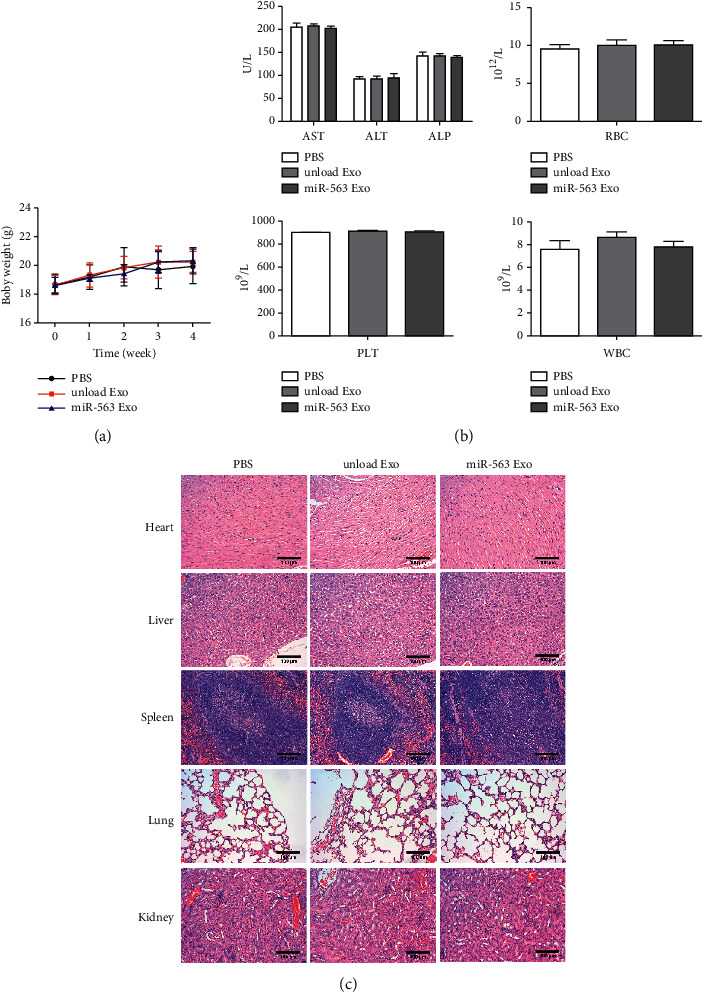
*In vivo* safety evaluation of miR-563 loaded engineered Exo. (a) Body weight of the test groups. (b) ALT, AST, ALP, WBC, RBC, and PLT changes of the test groups. (c) H&E staining of mice heart, liver, spleen, lung, and kidney sections. Scale bar, 100 *μ*m. ALT, alanine aminotransferase; AST, aspartate aminotransferase; ALP, alkaline phosphatase; RBC, red blood count; PLT, platelets; WBC, white blood count. The values were presented as mean ± SD (*n* = 3).

The toxicity is another key parameter of an excellent delivery vehicle. To evaluate whether miR-563 loaded engineered Exo was safe, we assessed the systematic toxicity of miR-563 loaded engineered Exo in healthy BALB/*c* mice at a dosage as previously described. Compared with PBS and unloaded Exo group, no change in the body weight was observed (Figure 7(a)). We further explored the potential pathological damage of Exo to organs. Different biochemistry indexes were detected, including the liver function indexes ALT, AST, and ALP (Figure 6(b)), and the results suggest that miR-563 loaded engineered Exo does not affect liver function. Furthermore, we also assessed the hematological, WBC, RBC, and PLT counts. All the above parameters of miR-563 loaded engineered Exo treated group indicated no significant difference (Figure 7(b)). In addition, as shown in Figure 7(c), heart, liver, spleen, lung, and kidney staining showed that there were no histopathological abnormalities or lesions compared in these organs, suggesting that there was no pathological change caused by miR-563 loaded engineered Exo. The above results demonstrated that miR-563 loaded engineered Exo did not influence the blood system and major organs in healthy mice.

## 4. Discussion

With the constant development of nanotechnology for the treatment of cancers, nanomaterials have been widely used in various fields. Exosomes are natural macromolecular substance carriers between human cells, which affects cell-cell communication [21]. Exosomes are stable, so it can improve the half-life of drugs in the process of blood circulation. The special lipid composition of exosomes stimulated the fusion between exosomes and target cells and also enhanced the cell-cell communication [22, 23]. There were many studies on the functions of exosomes for delivering gene drugs for the treatment of tumors [24]. For example, miR-317b-5b loaded engineered exosomes had the antitumor functions by delivering drug molecules [25]. In this study, we obtained miR-563 from GeneCard, which has been founded to play a tumor inhibitory role in the progression of LC by targeting related oncogenes by Zhang et al. [19].

We first assess the miR-563 by RT-PCR and ISH in LC tissues and adjacent tissues. We found that miR-563 levels were downregulated in the cancer tissues. Next, we successfully constructed miR-563-loaded engineered Exo to explore whether the miR-563 has the potential for the treatment of LC. Our findings suggested that miR-563 loaded engineered Exo were specifically uptaken by A549 cells. Internalization of the miR-563 loaded engineered Exo in tumor cells influenced the cell behaviours. Our results demonstrated that miR-563 loaded engineered Exo was able to inhibit the tumor cell growth *in vitro*. Furthermore, the tumor inhibitory effect of miR-563 loaded engineered Exo was evaluated *in vivo*. The lowest tumor volume and weight were observed in mice treated with prepared Exo compared with the PBS and unloaded exosomes, which showed significant antitumor efficiency of miR-563 loaded engineered Exo. *In vivo* studies also showed the effective delivery of miR-563 to tumor tissues and induced tumor cell apoptosis mediated by miR-563 loaded engineered Exo.

Furthermore, after fine modification, nanodrug carrier can combine with cancer cell membrane, microenvironment, or cytoplasm, so as to deliver high concentration drugs to targeted cancer cells and reduce the toxicity to normal cells [26–28]. In order to explore whether the miR-563 Exo is safe to other cells, we have to assess the liver function and hematological, WBC, RBC, and PLT of the mice. There was no significant difference between miR-563 Exo treatment group and the other group. H&E staining also found that the engineered exosomes had no toxicity on major organs. Our results demonstrate that miR-563 loaded engineered Exo can be used as a feasible nanocarrier to deliver drug molecules and can play its antitumor role in LC. In conclusion, our study indicated that miR-563 loaded engineered exosomes have potential for clinical application. miR-563 loaded engineered exosomes can be a novel potential therapeutic target for lung cancer treatment.

## Data Availability

All data generated or analyzed during this study are included in this published article.
